# Microstructure and phase composition of bronze Montefortino helmets discovered Mediterranean seabed to explain an unusual corrosion

**DOI:** 10.1038/s41598-021-02425-6

**Published:** 2021-11-26

**Authors:** Francesco Armetta, Maria Luisa Saladino, Antonella Scherillo, Eugenio Caponetti

**Affiliations:** 1 Dipartimento Scienze e Tecnologie Biologiche, Chimiche e Farmaceutiche - STEBICEF, Università di Palermo, Viale delle Scienze Ed. 17, 90128 Palermo, Italy; 2 Science and Technology Facility Council, ISIS Neutron and Muon Source, Didcot, OX110QX UK; 3 Labor Artis C.R. Diagnostica S.R.L., Via Celona, 90145 Palermo, Italy

**Keywords:** Chemistry, Materials science

## Abstract

Two Monterfortino helmets, recovered in the Mediterranean seabed, show unusual features with respect to the more common helmets of the same period and found in underwater environments. Hence, they were investigated by a multi-analytical approach, which allowed us to identify the compounds constituting the helmets and to make some considerations about their metallurgy, although all the metal was converted to degradation products. The helmets, originally made in bronze, have maintained their original shape because of copper sulphides formation. The observed differences in composition between the two helmets were attributed to the position modification, of one of them, into the seabed along centuries. For the first time, a microstructural investigation permits to reconstruct the history of the aging processes involved in the total oxidation of roman bronze helmet metal.

## Introduction

In the last decades a rich frame of discoveries has been made around the entire Mediterranean Sea. A wide series of wrecks, dating back to the II millennium BC until recent time, has been found in the Mediterranean seaboard^1^. Among wrecks, we can distinguish a fairly wide variety of ship typologies as well as cargo quality and quantity^2^. Amphorae, anchors, parts of boat wood are probably the most common findings, but in some cases rare objects have been found and represent precious case studies both for archaeologists and scientists who try to understand the history and the manufacture of the artefacts. In many cases most of the requested information are focused on the production and/or the usage of the items^3,4^, in some cases the interest is mostly spent to understand the behaviour and the effect of the permanence of the object in the sea environment^5,6^. Sea water, sand, microorganisms and marine wildlife can contribute to the object aging with a wide series of chemical, physical and biological degradation involving corrosion and concretion formation. The degradation of metallic archaeological findings in underwater environments has been thus object of several studies, which tried to understand how environmental microchemical parameters can affect and direct the disease and the formation of corrosion products. Several papers described the investigation of metals items recovered in the seabed after many years of aging^3,7^. Some of these studies focused on the understanding of the alloy corrosion processes influenced by the environment conditions^8^, others regard the determination of corrosion products to establish the conservation state of the artefacts and the strategies to apply for their restoration and conservation^9–13^.

There are several interesting cases studio about the possibility to reconstruct the history of a bronze artifact trough the analysis of patinas^14^ i.e. Ingo et al. identified by a turquoise crust the interaction of a roman coin with phosphorous released by animal bones discarded into the ancient sewer^15^ or correlated the accumulation of metal on the Dancing Satyr with the marine context^16^.

**Figure 1:**
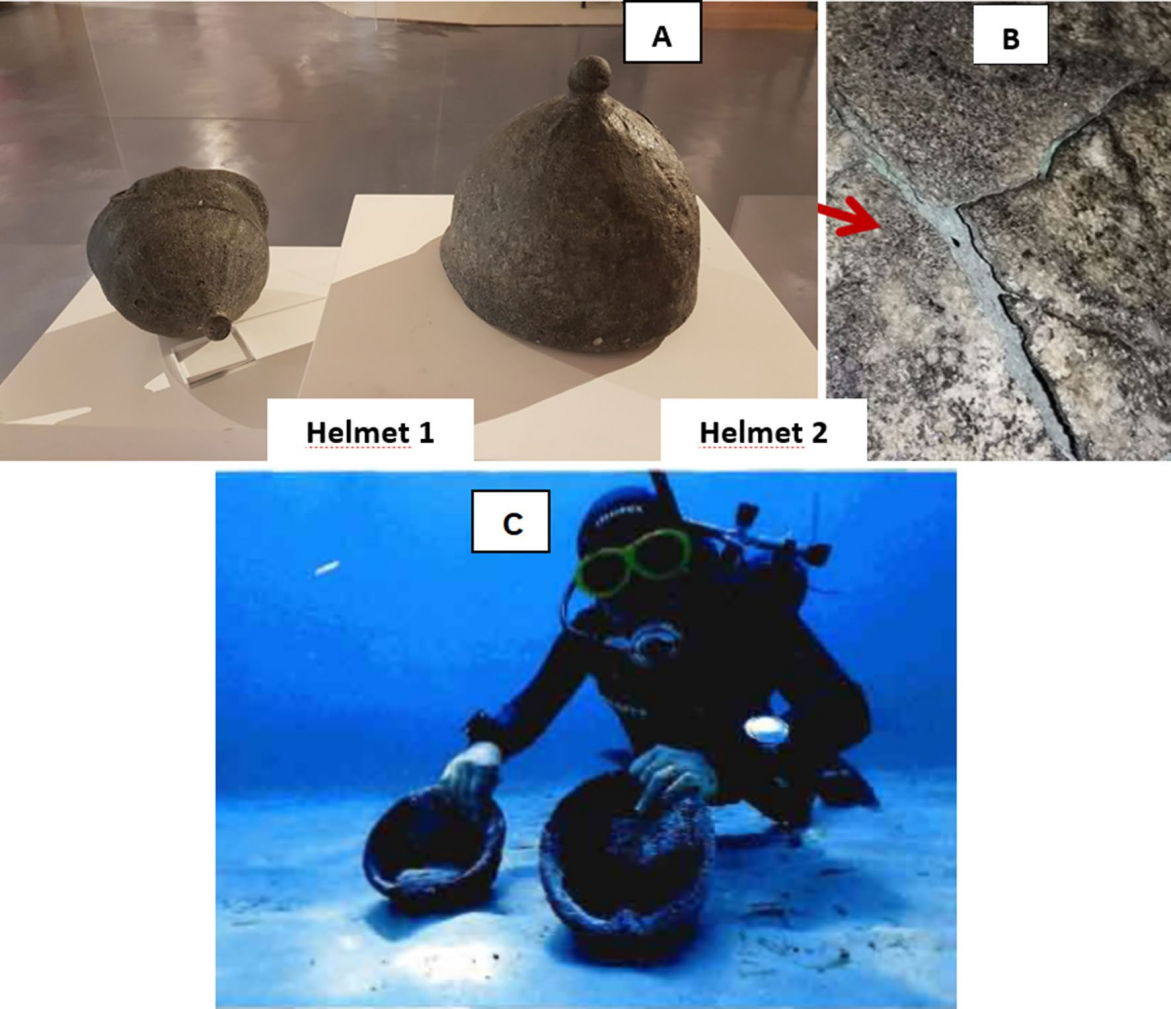
Photographs of the helmets in the museum exposition and detail of helmet 2 (**A**), detail of the surface of helmet 2 (**B**) and discovery (C)^18^.

In this paper, we report an investigation about two unusual helmets found in the seabed of Capo San Vito (Trapani, Italy) in an area where a Spanish galleon, guns and, other finds were discovered roughly twenty years ago. The presence of a galleon shipwreck, dated to 1526 close to the two helmets reminded archaeologists that they came from the same period^17^. The helmets are currently exhibited in the Museo Archeologico Regionale Lilibeo—Baglio Anselmi of Marsala (Trapani, Italy) and are indexed as helmet 1 and helmet 2 (Fig. 1).

The wall blackish surface is heterogeneous and full of small white concretions entrapped inside a black matrix, few cracks are recognized in helmet 2 (magnification in Fig. 1B), finally the consistence of this material looks different from a metal stuff. Two cheek pieces are present in helmet 1 and only one in helmet 2. The cheek pieces, usually connected by hinges in order to make them movable, in both case are bended toward the internal part of the helmets and form one with the wall (Fig. 1B).

Few years ago, as a result of more in-deep observation, the helmets were classified as Montefortino-type. This type of helmets was widely used in the Mediterranean area from V century B.C. to I century A.D. and is characterized by a rounded tip (apex) in the top, two cheek-pieces on the sides, and a projecting wing at the rear to protect the wearer's neck^19^. About two hundred of these Montefortino helmets have been recovered from battle areas and tombs of italic ancient town. Archaeologists classified this huge number of objects in 12 different typologies on the bases of different geographical areas, style differences developed by smiths, and military rank^20^.

**Figure 2:**
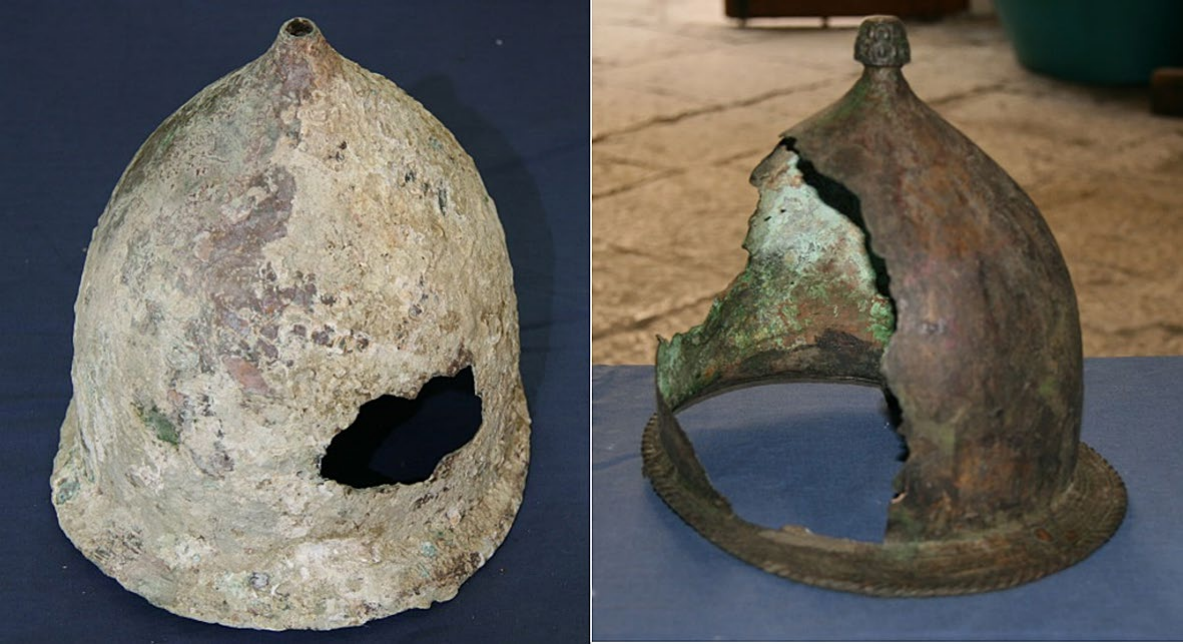
Example of Montefortino helmets recovered in the Egadi seabed after partial restauration.

The original difficulties in the attribution were due to their uncommon features respect to the ones scheduled as Montefortino-type helmets. The values of their weight (3.5–4.0 kg) and of the wall thickness (1.5–2.0 cm) are much higher than those of Montefortino helmets (~ 0.5 kg and ~ 2 mm, respectively) recovered in underwater environments such as the ones reported in Fig. 2.

Thus, it is reasonable to think that an unusual degradation process modified the original aspect of the helmets making difficult the identification of their typology^21^.

The unusual features of helmets of Capo San Vito represent an interesting case study that requires to be deepened. The aim of this study was thus the determination of the composition of the two helmets in order to provide an explanation about their occurred unusual degradation and to reveal archaeometric details useful for their classification.

## Experimental

In a first stage we have undertaken a non-invasive investigation using both surface and bulk techniques. However, the obtained results were not enough to reach the goal and therefore, it was decided to carry out a micro-sampling on both helmets. First, X-ray Fluorescence spectroscopy (XRF) was applied for the determination of the surface elemental composition^22^ and Neutron Resonance Capture Analysis (NRCA) as well as Neutron Diffraction (ND) were applied for the determination of the bulk elemental composition and the crystalline phases composition, respectively. There are several studies regarding the application of neutron techniques to the study of archaeological finding, the high penetration power of neutrons makes them a formidable non-invasive probe for matter characterization^23–26^. In particular, neutrons have been widely used for the analysis of high-density materials such as metals, providing bulk information that cannot be obtained using X-Rays techniques.

On the basis of the obtained results, a more deepen investigation was undertaken by collecting 4 microsamples (~ 10 mg each) at different depth from the surface up to the median part of the wall thickness of each helmet by using a Dremel microdriller. Letters from A to D were used to identify samples from the surface to the inner part. Two photos of the sampling operation are reported in Fig. S1 of Support Information (SI). The collected powders were analysed by XRF Spectroscopy and X-Ray Diffractometry (XRD) in order to acquire the same information obtained on the surface by XRF and as mean value of the whole wall thickness of the helmets by NRCA, but at different depth from the surface. In addition, one erratic fragment, originated from the internal part of helmet 2, was analysed by Scanning Electron Microscopy (SEM) to observe the internal morphology.

*XRF* spectra were acquired by using a Tracer III SD Bruker AXS portable spectrometer. The irradiation by a Rhodium Target X-Ray tube operating at 40 kV and 11 mA and the detection of fluorescence X-rays by a 10 mm^2^ silicon drift X-Flash detector allow the detection of elements with atomic number Z > 11. A window of 3–4 mm in diameter determined the sampled area. Each spectrum was acquired for 30 s. The S1PXRF® software was used for data acquisition and spectral assignments. The fluorescence signal area was estimated once the de-convolution of the whole spectrum was performed by using the software ARTAX 7.

*NRCA* and *ND* investigations were performed at INES neutron diffraction beam-line, at ISIS Neutron Spallation Source (Rutherford Appleton Laboratory, STFC, UK). The beam-line geometry was developed for the analysis of cultural interest samples. Considering the presences of two check piece in helmet 1 and one in helmet 2, it was possible to perform the investigations only on the helmet 2 where the absence of one check piece makes accessible half of the shell of the helmet allowing the neutron beam to go through the helmet thickness.

INES beam-line is characterized by a good resolution power and it is equipped with an array of 144 ^3^He detectors grouped in nine banks and covering on the horizontal plane a scattering angle ranging from 11.6° to 170.6°. Thus, it is possible to apply the Rietveld refinement to a set of nine independent diffraction patterns to obtain the quantitative phase composition of the investigated sample. Only the first eight banks diffraction patterns were used excluding the forward-scattering one, since its d-spacing coverage is not useful for the searched phases. INES beam-line is also equipped with an Yttrium–Aluminium–Perovskite (YAP) gamma scintillation detector installed above the sample tank; it allows performing NRCA analysis, which provides the bulk elemental composition. The neutron beam spot was 10 × 20 mm. Several points were chosen to test the helmet compositional homogeneity. Each analysed point was irradiated for about 10 h to reach an integrated proton beam current of about 200 mAh.

*XRD* patterns were acquired by a Philips PW 1050/39 diffractometer on the powdered samples obtained by the drilling procedure. The diffractometer operates in the Bragg–Brentano geometry using Ni-filtered Cu Kα radiation (λ = 1.54056 Å) in the 2θ range 5°–75° with a step of 0.05° and a time for step of 5 s. X’pert HighScore® Software was used for the qualitative interpretation of the patterns while MAUD^27^ was used for the Rietveld analysis.

*SEM* observations were performed by a FEI Company, Hillsboro, OR Quanta 200 instrument equipped with an Energy Dispersive Spectrometry (EDS) system. The small fragment was stuck onto an aluminum stub by means of double sided adhesive conductive carbon tape and was observed without a metallization process.

## Result and discussion

### Non invasive investigation

#### XRF

**Figure 3:**
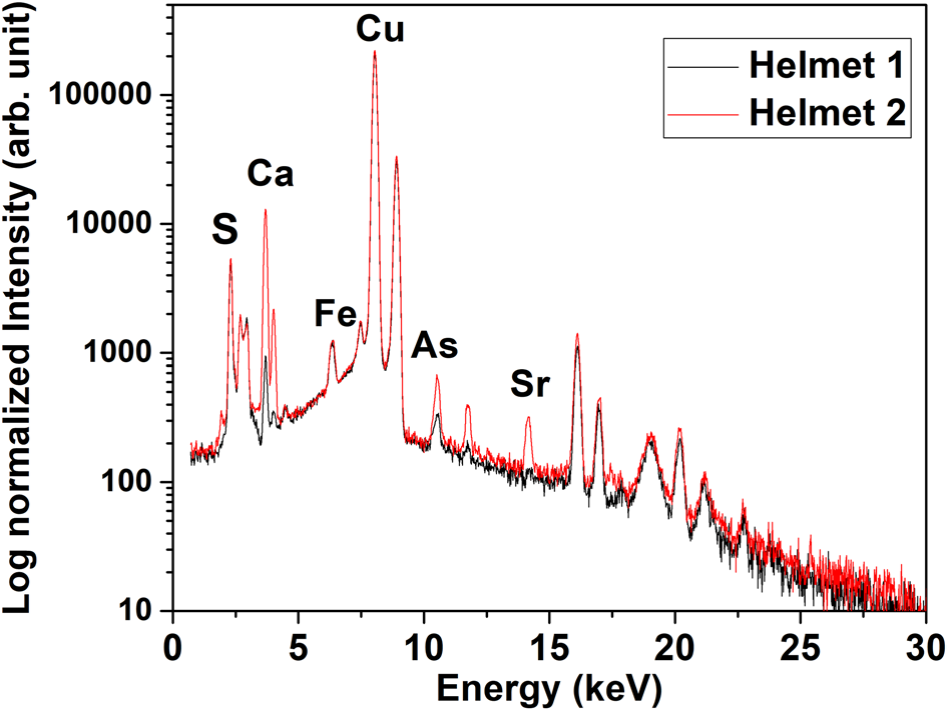
Two representative X-Ray fluorescence spectra of the two helmet surfaces.

For all analysed spots of each helmet the XRF spectra are similar. As an example, two representative spectra of the two helmets are reported in Fig. 3.

The presence of high copper peaks was observed together with small peaks of iron, arsenic, calcium, strontium and sulfur. The not indexed peaks come from instrumental contribution or from elements already indexed. Copper and arsenic are elements already present in the alloy, iron could be also originated by a surrounding corroded iron objects that have produced ions that have been incorporated into the patina, the origin of calcium and strontium is due to environment source contaminations, whereas the sulfur presence is unusual.

#### NRCA

**Figure 4:**
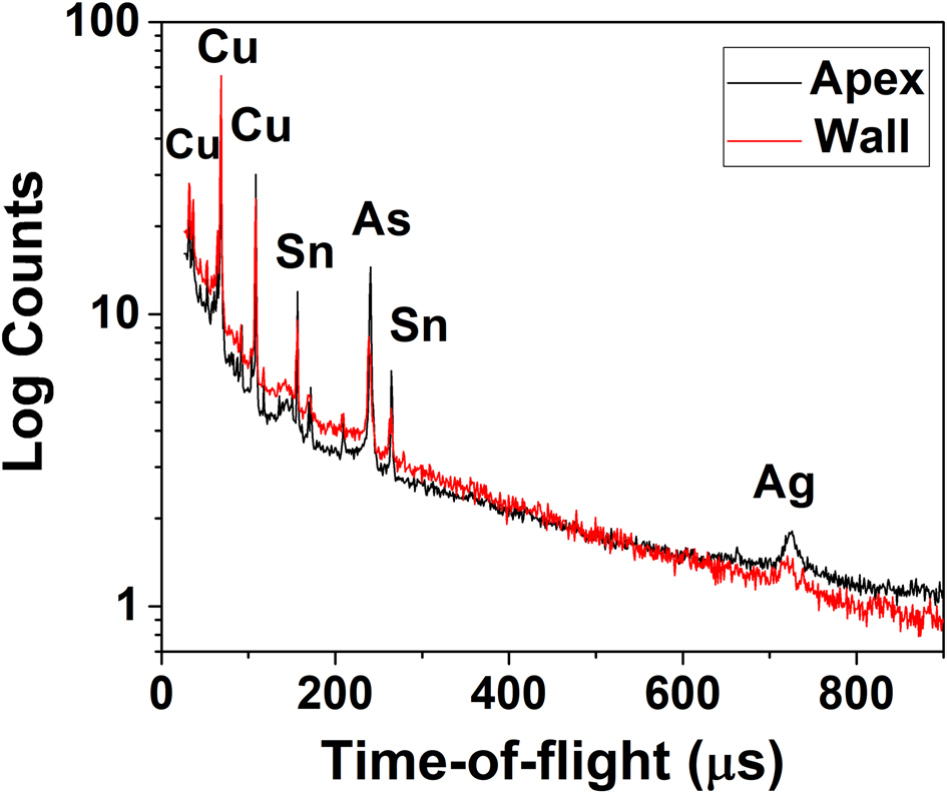
NRCA spectra of two representative spots of helmet 2.

The bulk elemental composition obtained from helmet 2 by NRCA reveals the presence of copper, tin, arsenic and silver. Two representative spectra, acquired the first one on the wall and the other on the Apex, are reported in Fig. 4.

No substantial difference is observed in the spectra of the two analysed spots indicating that the helmet is quite homogeneous. The presence of intensive copper peaks together with some small peaks of arsenic, tin and silver is observed. Tin and silver were not detected by the XRF analysis indicating that these two elements are mainly localized in the inner part of the artefact. It is also worth to note that iron and sulphur peaks observed in XRF spectra are not present because their resonance values are out of the measurable range at INES beam-line.

The simultaneous presence of small amount of arsenic and silver suggests the use of not completely roasted sulphides ores (i.e. enargite Cu_3_AsS_4_) and rather poor refining practices^28^. This finding related to the metallurgy of the helmet is important because it can be an indication of the use of copper sulphide ores as mineral source^29^ that is common for roman bronze artefacts^30^ another reasonable explanation regards the recycling of much more older alloys like arsenical bronze, which usually contains minor amount of silver^31^ and or mixing with some copper-silver scrap metal^29^. Usually the presence of silver can be correlated to the lead source but in our case the lead is almost absent.

The composition of the apex provides some details about the use of the helmet. It is well known that the helmet of special forces was bedecked with plumage and in this case the hollow apex was filled with lead to hold the crest pin securely in place^19^ therefore, the absence of lead indicates that the helmet belonged to common soldiers.

#### ND

**Figure 5:**
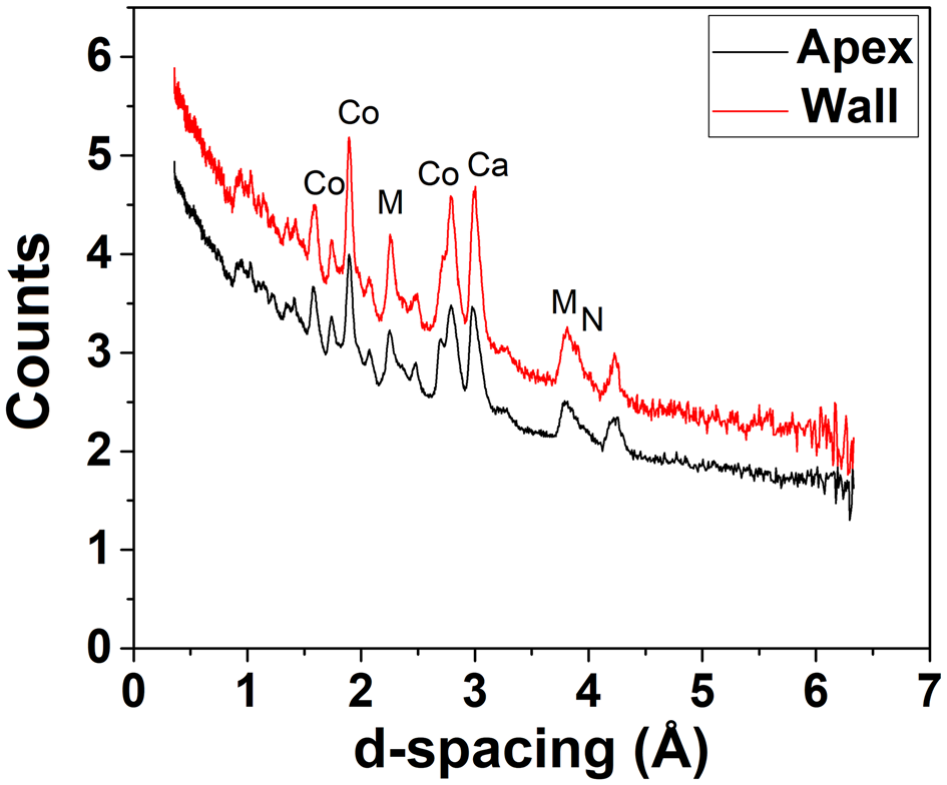
Two representative neutron diffraction patterns of helmet 2 (*Co* Covellite, *Ca* Calcite, *N* Nantokite, *M* Mushistonite).

The ND obtained patterns performed on several points of helmet 2 can be described in terms of wide peaks and a high baseline. For some of the spots the peak intensities are low, but they do not evidence significant differences. The patterns contain the same peak sequences with small differences in the relative intensities, confirming the helmet homogeneity. Two representative patterns acquired, the first one on the wall and the other one on the Apex, are reported in Fig. 5.

The highest peaks were assigned to covellite (CuS, Co), compound found on few ancient bronze objects that were found buried in seabed or lake sediments^32^. Nantokite (CuCl, N) is a common corrosion product for environments rich in chlorine. The presence of calcite (CaCO_3_, Ca) can be associated to the white shell-fish inclusions observed in the surface or to the precipitation of calcium carbonate from the CO_2_ dissolved in the seawater. Finally, the peaks indexed with M were assigned to mushistonite ((Cu, Fe)Sn(OH)_6_), an “exotically” hydroxide complex. In most of the papers, concerning neutron diffraction studies of bronze artifacts, the peaks of alloy lattices are well defined and provides information about the composition and the processing of the alloy^33–35^. It is interesting to note that, in the present work, the characteristic peaks of metallic copper phases are absent indicating that the whole original metals were completely converted into degradation products. It is interesting to notice that the identification of covellite and calcite, as main phases, is in agreement with the measured density value 4.5 and 4.8 g cm^−3^ and are very far from the values of c**o**pper or bronze (8.94 and 8.8 g cm^−3^, respectively). These values are instead similar to the ones of calcium carbonate and sulfate (~ 3 g cm^−3^) and very close to the one of c**o**pper sulfides (~ 4.5 g cm^−3^).

### Micro-sample powder investigation

#### XRF

**Figure 6:**
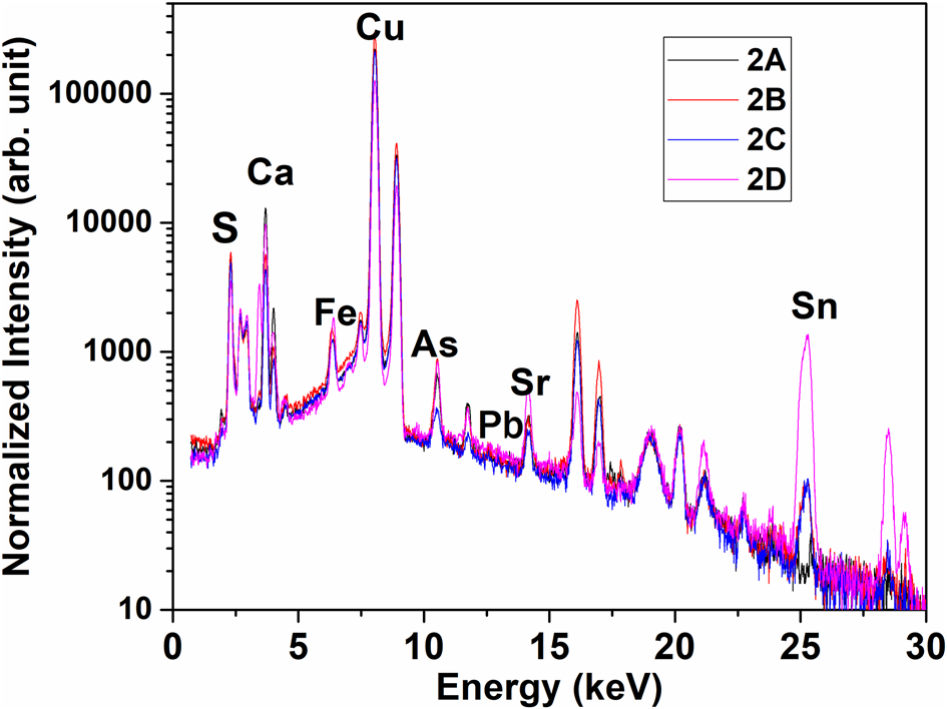
XRF spectra of the powder samples of helmet 2.

The spectra acquired on the four powder micro-samples for each helmet, collected at different depth from the surface, are reported in Fig. 6.

In addition to NRCA, XRF investigation reveals the presence of sulphur, calcium, iron strontium and lead, while silver signals are not observed. Moreover, XRF provides a further information about the elements localization along the helmet wall thickness.

The most interesting result regards the mean value of the calculated copper/sulphur peak area ratios for all the samples (70). This value is a bit smaller than the one obtained from the CuS powder (80), indicating that the copper/sulphur atomic ratio, in our samples, is very near to one. This means that almost all the copper has been transformed in copper sulphides that they are pervasive.

The peak intensity increases significantly for tin and slightly for arsenic from surface to the inner part. This variation indicates that a flow of ionic species from one region to another took place suggesting that corrosion occurs with a potential difference in the reaction process^19^.

It is known that during bronze degradation tin tends to move away in agreement with the low amount found in the external samples (destannification). In addition, the ionic tin diffusivity in the oxidised copper matrix decreases^36^ justifying its higher amount in the internal sample (D) and suggesting that tin could be the second main element constituting the artefact. This find agrees with the classification as Montefortino type helmet that was usually made of a copper-tin alloy, even if there are some iron Montefortino helmets produced by Celtic manufacture^19^. However, it has to be considered that the small tin peak could be due to a low amount of tin was in the original alloy.

Finally, lead was detected along the depth profile and its low amounts suggest that it wasn’t voluntary added to the original alloy. The reasonability of this hypothesis is reinforced by the fact that the helmet manufacturing was usually performed by a hammering process^19^ because lead, being not soluble in copper alloys when the temperature drops to the ambient one forms dendrites clearly separated from the copper phase, these lead islands penalise the mechanical resistance and handwork production as suggested by Griesser^37^ and Di Turo^38^.

#### XRD

**Figure 7:**
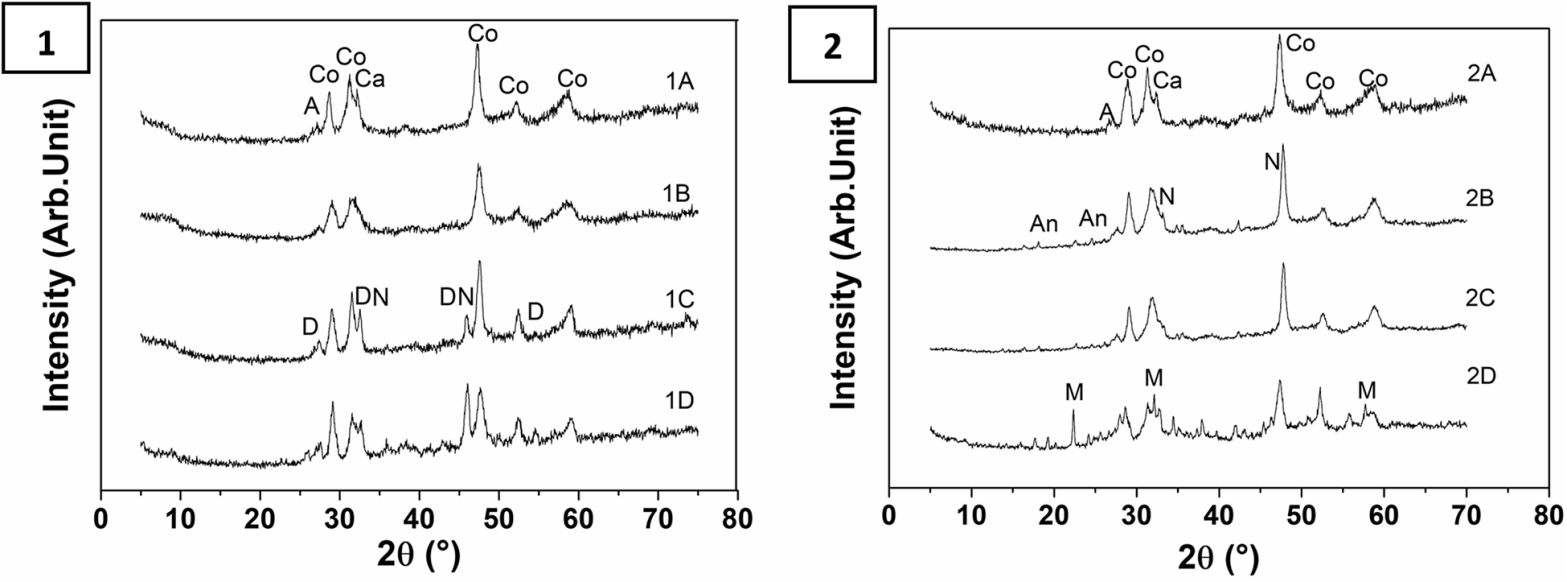
XRD patterns of the powder samples of helmet 1 (#) and helmet 2 (#), where # indicates the sample position from surface. (*Co* Covellite, *D* Digenite, *Ca* Calcite, *A* Aragonite, *N* Nantokite, *M* Mushistonite, *An* Antlerite).

Patterns of the four powder samples for each helmet are reported in the Fig. 7.

XRD patterns are noisy because of the low powder amount collected by using a micro-driller; it was decided to collect a so small amount of powder in order to reduce the analysis invasively, nevertheless the pattern resolution was good enough for allowing the phase identification. The knowledge of the elemental composition derived from XRF analysis helped us to identify some possible phases. The Rietveld refinement^39^ was applied to define the hypothesized phases by checking the peak mutual intensities and to determine their relative amounts. Covellite, Digenite, Antlerite, Nantokite (copper corrosion products), Mushistonite (tin degradation product), Calcite and, Aragonite (shellfish shell inclusions or calcium carbonate precipitation) were identified. Their relative amounts slightly vary from one sample to another.

Comparing with neutron diffraction measurements, it is interesting to notice that the sampling allows us to define the spatial collocation of each phases along the wall thickness. In detail, Mushistonite ((Cu, Fe)Sn(OH)_6_) was identified in the most internal sample (2D) of helmet 2 in agreement with the higher amount of tin detected by XRF. Mushistonite is similar to the schoenfliesite (MgSn(OH)_6_) that was found by Ingo et al.^9^ in a corroded bronze artifact containing sulphides. Only a few cases of tin corrosion products in bronze artifacts degraded in seawater are reported in literature. In one case-study, MacLeod et al. observed that during bronze corrosion under partially aerobic condition, tin reacts forming the oxide sulphate Sn_3_O_2_SO_4_^40^. Other studies^41^ found that tin oxidation in anaerobic environment produces cassiterite (SnO), a product always present together with cuprite (CuO) because of the similar redox potential (Eh) which drives their formation. Finally, a small amount of antlerite was found in samples 2B, 2C and 2D.

In order to highlight the behaviour of copper corrosion, we calculated the phase composition considering only the copper phases. Results are reported in Table 1.

**Table 1:** Copper phases composition (wt.%) of depth profile samples of the two helmets.

	Nantokite (CuCl), wt%	Digenite (Cu_1.9_S), wt%	Covellite (CuS), wt%	Antlerite Cu_3_ (OH)_4_SO_4_, wt%
1A	0	0	100	0
1B	0	0	100	0
1C	2	22	76	0
1D	2	56	42	0
2A	0	0	100	0
2B	3	0	93	4
2C	2	0	94	4
2D	5	0	89	6

The presence of nantokite, in the internal samples of both helmets, is an evidence that the corrosion started with the chlorine ions and it guided the formation of the other corrosion products.

It is well known that the presence of nantokite is detrimental to the stability of the bronze artefacts, since it activates the most dangerous form of post burial corrosion, commonly known as “bronze disease”^21^. The related cyclic reaction, involving oxygen and atmospheric humidity, disfigures the surface and disrupt the artefact. This cycle in anaerobic conditions is not favoured and others degradation processes occur. Usually, the analysis of copper based objects buried in seabed revealed the presence of cuprite (CuO), atacamite (Cu_2_Cl(OH)_3_) which cause the degradation of the metal and the formation of detachments^42^.

In our case, due to the presence of nantokite, it is reasonable to assume that the corrosion started with the formation of copper(I) chlorine but probably continued with the formation of copper sulphides.

It is known that the formation of the whole composition ranges of copper sulphides, from covellite to chalcocite (Cu_2_S), is favored by high concentration of sulphides and pH values ranging from 5 to 8 as reported in the Pourbaix diagram. Sulphide ions, whose formation can occur in anaerobic condition by the action of sulphate reducing bacteria (SRBs), can react with the oxidised copper^19,32,43^. During this interesting biological assisted corrosion process, bacteria participate in different steps of corrosion process thorough their enzymatic systems. The hydrogenases are able to depolarize the metallic surface to solubilize the metal and the produced electrons move to sulfate which is reduced in sulfur end involves the dissolution of the metal. At the same time the excretion of extracellular polymeric substances (EPS) improve the dissolution. The biofilm developed by D. desulfuricans at the metal surface accumulates with exposure time forming a poor protection patina^44^. Two examples, were the sulphides are present in corroded bronze, came from two quite recent wrecks, *Wasa* (1628 in Stockholm Habour)^45^ and *Mary Rose* (1545 near Portsmouth)^46^. In these cases, copper sulphides formation was justified by a biological activity favoured by the presence of organic matters due to the pollution of sewage and waste organic materials. Nevertheless, copper sulphides were found in copper objects of the *HMS Association* wreck (1707 Scilly Isles in UK)^47^, in a not polluted area where the anaerobic conditions were due to the sand covering.

Considering that the helmet discovery site is located in a not polluted sea area, we can assert, such as in the case of *HMS Association* wreck, that our samples spent most of the time buried under the sand.

Aragonite is often found as constituting mineral specie of the patina grown on bronzes retrieved from the sea water^11^ and it could be considered an interesting environmental marker. In fact, even if it is an unstable polymorph of calcium carbonate at room temperature and atmospheric pressure, its formation can be explained considering the nucleation mechanism proposed by Sun et al.^48^. They found a synergic effect of local pH values and Mg^2+^ ions presence which favour the formation of this metastable phase respect to calcite ones. The simultaneous presence of aragonite and calcite can be an indication of a modification of environmental parameters along centuries.

In helmet 1, together with nantokite, are present digenite and covellite respectively the lower and the higher oxidation copper sulphide. As can be observed in the Pourbaix diagram^13^ for copper degradation in seawater, both chlorine and sulphide (I) formation take place at similar pH values justifying the simultaneous presence of these two compounds. We can assert that the corrosion process, that started by the chlorine ions, continued with the formation of digenite first and covellite later. Both sulphides are present in the internal samples (1C and 1D) while covellite is the only phase in the more external ones (1A and 1B). This implies that, after some time, the helmet was subjected to a higher Eh (Pourbaix diagram)^49^ that can be due to the exposition of the artefact to an oxygenated environment. In the case of a burial artefact in sea water these conditions can be justified supposing that the helmet, for some reasons at certain time, was uncovered by the sand and spent some time on the seabed surface.

In the case of helmet 2 covellite is the only sulphide phase present in all the samples together with small amounts of antlerite in the internal samples (2B, 2C and 2D). The presence of the two copper II compounds could indicate that digenite was totally converted being the helmet subjected for a longer time to stronger oxidising conditions. This means that helmet 2 spent more time on the seabed surface uncovered by the sand.

## Helmet shape conservation

Once the helmet corrosion process has been explained, it is possible to hypothesize the reasons why the helmets could maintain their original shape. In the first stage, during the formation of copper (I) species, the retention of the shape of the helmets can be justified by an epitaxial relationship between bronze surface and corrosion products^19^ since it is known that they may growth by epitactic transformations^50^.

In the second stage, the transformation of copper(I) species into copper(II) ones took place in a solid-state reaction. Usually this type of reactions take place thought topotactic transformations that imply the loss of the original lattice structure. An example of topotactic growth is the transformation of copper sulphide into copper sulphate crystals^19^ that implies a huge change of lattice and therefore the loss of the shape. The retention of the shape in the second stage suggests that during the solid-state transformation only a slight alteration of the crystal lattice took place avoiding, strong damage of the corrosion layer. It is interesting to note that the presence of fissures, in the upper part of helmet 2, could be related to the presence of the small amounts of antelerite.

### SEM–EDS

**Figure 8:**
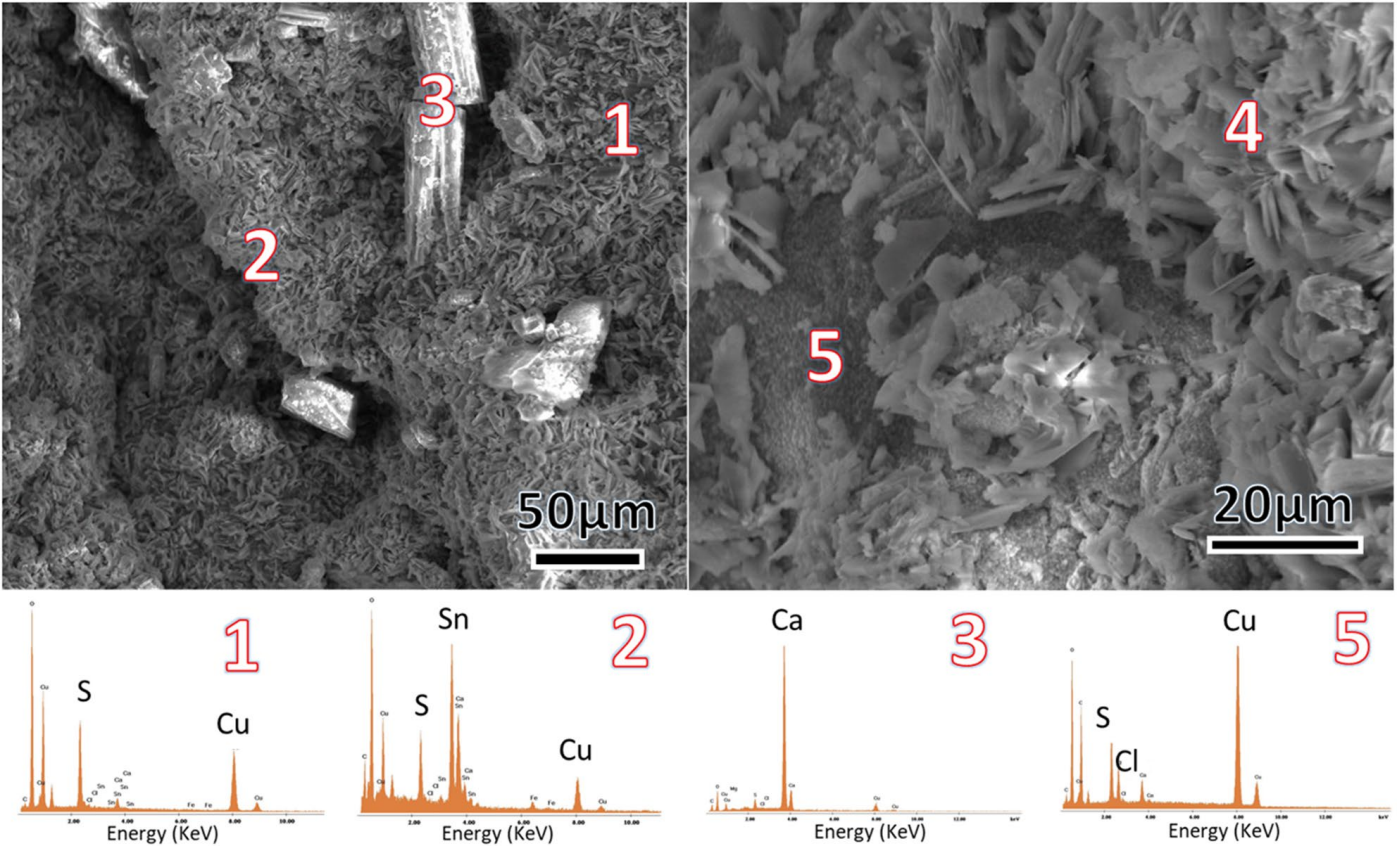
SEM micrographs of a small fragment detached from helmet 2 and EDS analysis of indicated spots.

Two representative micrographs of the small fragment detached from the internal part of a crack of helmet 2 are reported in Fig. 8.

The micrographs highlight the complex structure of helmets matrix, where crystals of different size and morphology constituting the artefacts can be observed. The numbers indicate the spots were EDS spectra were performed. The EDS spectra reported in Fig. 8 help us to identify the different phases forming the sample. The matrix is mainly composed by plate like crystals (points 1 and 4, just one spectrum is reported), the elemental analysis essentially reveals composed by sulphur and copper, ascribable to the covellite that according to XRD results constitutes the majority of the sample. It is interesting to notice that the size of the plate like crystal thickness (in the order of nm) justifies the XRD peak broadening of covellite phase. EDS spectrum of the small crystals in point 2 reveals the presence of tin and oxygen that can be related to the mushistonite phase. The small grains (point 5) under the plates like crystals constituted by copper and chlorine, are ascribable to the nantokite. EDS spectrum in point 3, containing the peaks of calcium together with the ones of oxygen and carbon, indicates the crystals are attributable to calcite providing an example of the ubiquitous presence of shell-fish fragment entrapped in the covellite matrix. It is reasonable that the high shell-fragment number of seawater organisms were entrapped during the sulfides growth.

## Conclusion

This paper reports the results of a study on the two unusual helmets that were discovered in Mediterranean seabed near Capo San Vito (Trapani, Italy). In comparison with the more common Montefortino-type helmets, the weight is much higher, the wall thickness is larger, and the density is significantly lower than that of metals. The reason why we undertake this study was the attempt to give a possible explanation of the processes that caused their unusual corrosion process and the maintaining of the original shape, as well as to find evidences, which may confirm their attribution.

The two helmets were investigated by means of the combined use of surface and bulk techniques by an approach using first not-invasive and then micro-invasive analysis that allowed us to obtain a deep profile composition of the phases.

In the complex structure of helmets matrix, crystals of different size and morphology were observed.

The most surprising outcome of the study was the absence of the pristine metals constituting the original helmet alloy and its conversion in copper sulphides.

The almost complete transformation of the original alloy into copper sulphides represents an unusual case of seawater degradation. Due to the presence of nantokite, in the internal samples of both helmets, it is reasonable to assume that the corrosion started with the formation of copper(I) chlorine.

In helmet 1 the presence of digenite, the lower oxidation copper sulphide, implies that the corrosion process, that started by the chlorine ions, continued with the formation of digenite, justified by anaerobic and sulphide rich conditions that are usually founded in polluted areas, but that in the present case can be attributed to the sand covering.

The presence of covellite implies that, after some time, the helmet was subjected to a higher Eh that can be justified supposing that the helmet was uncovered by the sand and spent some time on the seabed surface. In the case of helmet 2 the presence of covellite and antlerite indicates that digenite was totally converted and thus, that it spent more time on the seabed surface uncovered by the sand.

The explanation of helmet corrosion process makes possible to hypothesize the reasons why the helmets maintained their original shape. The initial formation of copper(I) species (nantokite and digenite), through an epitactic transformation, maintained the helmets shape. The subsequent formation of copper(II) species in the solid-state topotactic transformation caused a slight alteration of the crystal lattice avoiding damage of the corrosion layer.

It was also possible to hypothesize some aspects of the metallurgical process of the helmet manufacture and to assert that they were built in bronze, all information that agree with the classification as Montefortino type.

The proposed approach was evaluated as the best solution to extract additional information to the ones obtained by the non-invasive techniques, avoiding a more invasive cross section preparation that is usually necessary in order to perform a metallographic investigation.

## Supplementary Information

Supplementary Information.
