# Supplementary material for: Microstructure and phase composition of bronze Montefortino helmets discovered Mediterranean seabed to explain an unusual corrosion
Source: Sci Rep. 2021 Nov 26;11:23022. doi: 10.1038/s41598-021-02425-6 (PMC8626503; doi:10.1038/s41598-021-02425-6)
Supplement: Supplementary file 1 — Supplementary Information. [file 41598_2021_2425_MOESM1_ESM.docx]

**Microstructure and phase composition of bronze Montefortino helmets discovered Mediterranean seabed to explain an unusual corrosion**

Francesco Armetta ^1,*^, Maria Luisa Saladino ^1^, Antonella Scherillo ^2^, Eugenio Caponetti ^3^

^1^ Dipartimento Scienze e Tecnologie Biologiche, Chimiche e Farmaceutiche - STEBICEF, Università di Palermo, Viale delle Scienze Ed. 17, Palermo I-90128, Italy

^2^ Science and technology Facility Council, ISIS Neutron and Muon Source, OX110QX, United Kingdom

^3^ Labor Artis C.R. Diagnostica S.R.L., Via Celona, Palermo I-90145, Italy

** Corresponding author: francesco.armetta01@unipa.it*

**Support information**

**
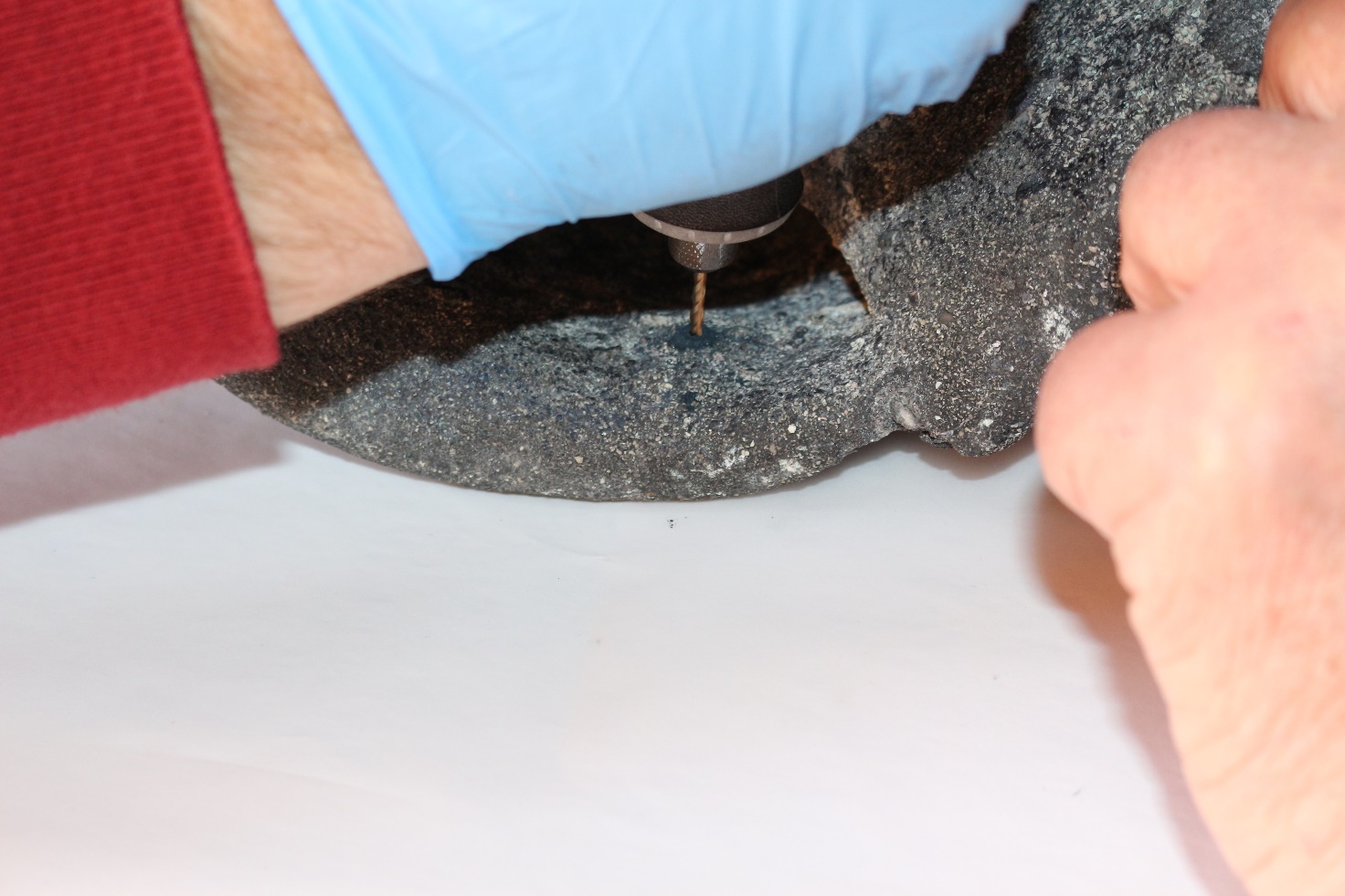
**

**
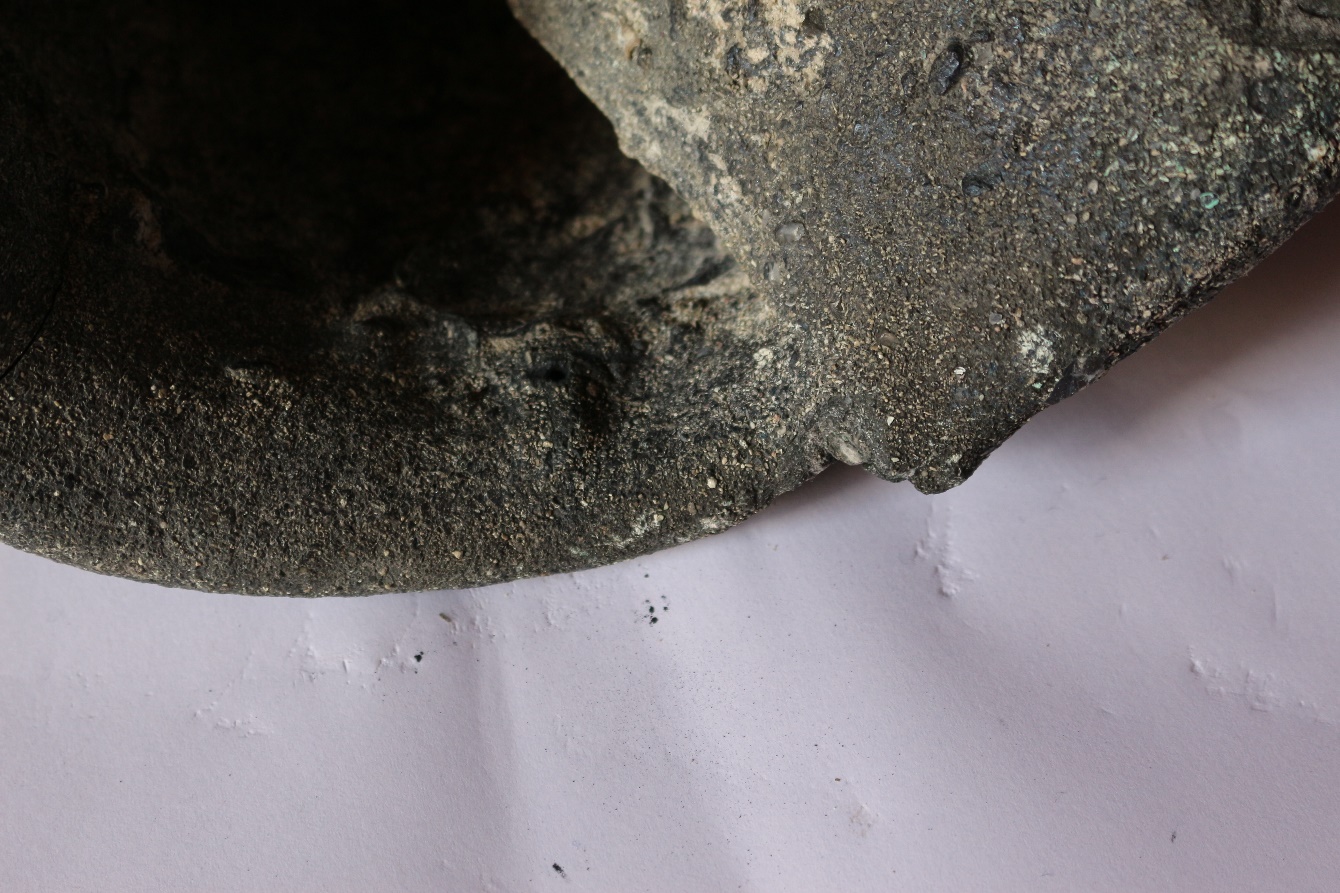
**

**Figure S1. Photographs of the helmet sampling operation**
